# Supplementary material for: Exploring functional microbiota for uranium sequestration in Zoige uranium mine soil
Source: Microbiol Spectr. 2025 Apr 16;13(6):e02517-24. doi: 10.1128/spectrum.02517-24 (PMC12131822; doi:10.1128/spectrum.02517-24)
Supplement: Supplemental material — Fig. S1 and S2. [file spectrum.02517-24-s0001.docx]

**Supplementary materials for:**

**Exploring functional microbiota for uranium sequestration in Zoige uranium mine soil**

Xiang Wang^1^, Xu Zhang^3^, Yanxia Wei^1^, Aixia Lu^1^, Jian Zhou^1,2,^*, Guiqiang He^1,2,^*

^1^ College of Life Science and Engineering, Southwest University of Science and Technology, Mianyang, Sichuan 621010, China;

^2^ Fundamental Science on Nuclear Wastes and Environmental Safety Laboratory, Southwest University of Science and Technology, Mianyang, Sichuan 621010, China;

^3^ College of Biomass Science and Engineering, Sichuan University, Chengdu 610065, China.

***Corresponding author:**

**Guiqiang He**

Mailing address: School of Life Science and Engineering, Southwest University of Science and Technology, Mianyang, Sichuan 621010, China.

E-mail: [guiqianghe@swust.edu.cn](mailto:guiqianghe@swust.edu.cn)

**Jian Zhou**

Mailing address: Fundamental Science on Nuclear Wastes and Environmental Safety Laboratory, Southwest University of Science and Technology, Mianyang, Sichuan 621010, China

E-mail: [zhoujian@swust.edu.cn](mailto:zhoujian@swust.edu.cn)

**Supplementary Material 1**

**Fig. S1** Soil samples from Zoige uranium mine in Sichuan, China. Soil samples were collected from four sampling points, including the East (E), South (S), West (W), and North (N) directions of the uranium mine center.


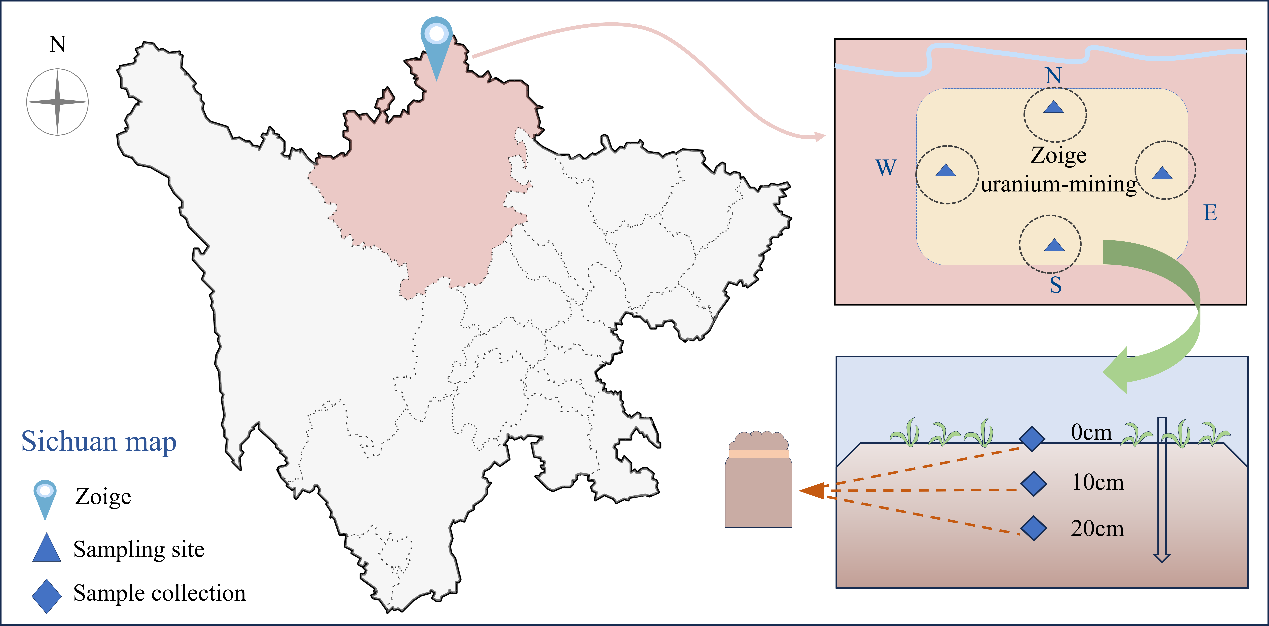


**Fig. S2** High-throughput sequencing results. (a) Sequence length distribution; (b) Rarefaction curves analysis for soil bacterial community based on Chao1 index.


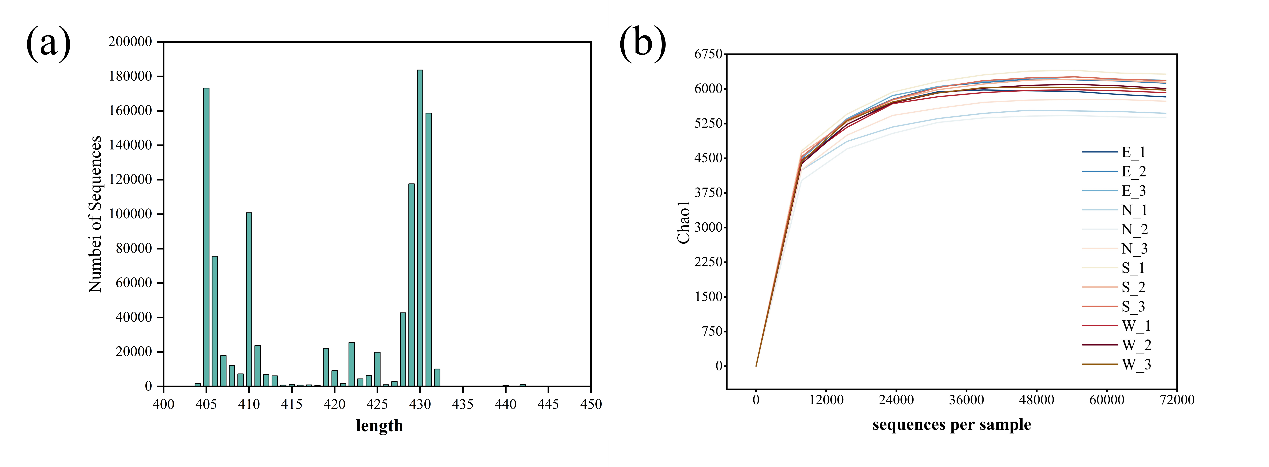


**Supplementary Material 2**

**Availability of data and materials:** The sequence data has been uploaded to the Surface Plasmon Resonance (SPR) database (Submission ID: PRJNA1101590). Sequence Read Archive (SRA) data can be accessed via <https://www.ncbi.nlm.nih.gov/sra/PRJNA1101590>.
